# Prokaryotic Diversity and Distribution Along Physical and Nutrient Gradients in the Tunisian Coastal Waters (South Mediterranean Sea)

**DOI:** 10.3389/fmicb.2020.593540

**Published:** 2020-12-01

**Authors:** Marianne Quéméneur, Malika Bel Hassen, Fabrice Armougom, Yosra Khammeri, Rim Lajnef, Amel Bellaaj-Zouari

**Affiliations:** ^1^Aix-Marseille Univ, University of Toulon, CNRS, IRD, MIO UM 110, Mediterranean Institute of Oceanography, Marseille, France; ^2^Institut National des Sciences et Technologies de la Mer, Salammbô, Tunis, Tunisia

**Keywords:** bacteria, archaea, bacterioplankton, diversity, Tunisia, Gulf of Gabès, seawater

## Abstract

Prokaryotes play an important role in biogeochemical cycling in marine ecosystems, but little is known about their diversity and composition, and how they may contribute to the ecological functioning of coastal areas in the South Mediterranean Sea. This study investigated bacterial and archaeal community diversity in seawater samples along the Tunisian coast subject to important physicochemical disturbances. The 16S amplicon sequencing survey revealed higher prokaryotic diversity in the northern Tunisian bays than in southeastern waters (Gulf of Gabès). The major taxonomic groups identified in all samples were *Alphaproteobacteria* (40.9%), *Gammaproteobacteria* (18.7%), Marine Group II *Euryarchaeota* (11.3%), and *Cyanobacteria* (10.9%). Among them, the relative abundance of *Alteromonadales*, *Prochlorococcus*, and some clades of *Pelagibacterales* (SAR11) significantly differed between the northern and the southern bays, whereas no difference was observed across coastal waters in the archaeal *Candidatus* Poseidoniales (MGII), *Synechococcus*, and *Pelagibacteraceae* (SAR11 clade Ia), for which no relationship was observed with the environmental variables. Both *Pseudoalteromonas* and *Alteromonas* levels increased with the increasing salinity, density and nutrients (NH_4_^+^ and/or PO_4_^3–^) gradients detected toward the southern waters, while the SAR11 clades Ib and IV and *Prochlorococcus*, decreased in the shallow, salty and nutrient-rich coastal waters of the Gulf of Gabès. *Rhodobacteraceae* was positively correlated with *Synechococcus* and chlorophyll levels, suggesting a relationship with phytoplankton biomass. The present study provides the first insights into planktonic prokaryotic community composition in the South Mediterranean Sea through the analysis of Tunisian seawaters, which may support further investigations on the role of bacterioplankton in the biogeochemistry of these ecosystems.

## Introduction

Prokaryotes are abundant in marine ecosystems, with number estimates of 1 × 10^29^ in oceans and concentration estimates of 10^3^ bacteria per microliter in surface seawater (Azam and Malfatti, 2007; Flemming and Wuertz, 2019). They are the most diverse metabolically and phylogenetically organisms on Earth (Oren, 2004). They also play a central role in biogeochemical cycles, marine food chains and global climate change (Shively et al., 2001; Cotner and Biddanda, 2002; Azam and Malfatti, 2007; Falkowski et al., 2008; Strom, 2008; Liu et al., 2010; Zehr and Kudela, 2011; Bao et al., 2018). Photosynthetic prokaryotes are responsible for a large proportion of the total primary production (Azam and Malfatti, 2007; Flombaum et al., 2013), while heterotrophs play an important role in the microbial loop by the remineralization of organic compounds (Carlson et al., 2010). Photosynthetic *Cyanobacteria* and photoheterotrophic *Alphaproteobacteria* of the order *Pelagibacterales* (also known as SAR11) are among the most abundant prokaryotes in marine waters (Chisholm et al., 1988; Morris et al., 2002; Carlson et al., 2009; Flombaum et al., 2013; Evans et al., 2015; Giovannoni, 2017). In the Mediterranean Sea, most studies have identified SAR11 as the dominant bacteria (ranging between 25 and 45% of the reported sequences and contributing to 36 ± 6% of total prokaryotic abundance; Laghdass et al., 2012), followed by other *Alphaproteobacteria* (belonging to the family *Rhodobacteriaceae*; Zaballos et al., 2006; Alonso-Sáez et al., 2007; Feingersch et al., 2010; Salter et al., 2015). *Cyanobacteria* (*Prochlorococcus* and *Synechococcus*), *Gammaproteobacteria* (e.g., *Alteromonadales*) and *Bacteroidetes* constitute the remaining of the dominant bacterial diversity with some divergences depending on depth, seasons, and distance from land (Estrada and Vaqué, 2014). Diverse archaeal communities have also been observed in the Mediterranean Sea water column and their structure also varied depending on depth, geographic areas, and seasons (Massana et al., 2000; Galand et al., 2010; Hugoni et al., 2013; Martin-Cuadrado et al., 2015). Marine planktonic *Archaea* are currently classified into four main groups belonging the phyla *Thaumarchaeota* (formerly Marine Group I archaea) and *Euryarchaeota* (Marine Groups II, III, and IV; Santoro et al., 2019). Although many studies have evaluated the spatiotemporal diversity of prokaryotes in several marine regions throughout the world, including the Mediterranean Sea (Ghiglione et al., 2005; Feingersch et al., 2010; Galand et al., 2010; Techtmann et al., 2015), their responses to environmental changes remain largely unknown in many specific coastal areas, especially along the southern coasts of the Mediterranean Sea, impacted by anthropogenic activities, climatic changes, and a complex water circulation pattern (Bel Hassen et al., 2009a; Coll et al., 2010; Zouch et al., 2018; Béjaoui et al., 2019).

The Mediterranean Sea is a semi-enclosed oligotrophic sea characterized by high variations in environmental conditions (e.g., temperature, salinity, current, and nutrient availability) leading to high change in biodiversity depending on depth, seasons, and location (Coll et al., 2010). The salinity, temperature and nutrient gradients are especially pronounced off the coasts of Tunisia, comprising the Strait of Sicily, which represent the natural conjunction between the western and the eastern Mediterranean basins (Khammeri et al., 2020). This eastern basin is the most oligotrophic area of the Mediterranean Sea, and their phytoplanktonic communities were mainly composed of ultraphytoplankton (<10 μm), which play an important role in the primary production (Bel Hassen et al., 2009b; Estrada and Vaqué, 2014; Khammeri et al., 2020). In the eastern Mediterranean basin, the Gulf of Gabès is one of the most productive Mediterranean area (Ayata et al., 2018), due to the favorable climatic conditions and the high nutrient availability, contrasting with the oligotrophic waters of the eastern basin (Béjaoui et al., 2019). Changes in picoeukaryotic assemblages, phytoplankton abundance and ultraphytoplankton composition have been largely studied in the shallow waters of the Gulf of Gabès (Bel Hassen et al., 2009a, b; Rekik et al., 2014; Hamdi et al., 2015; Bellaaj-Zouari et al., 2018). Recently, the ultraphytoplankton distribution have been evaluated by flow cytometry analysis along the Tunisian coast, showing a gradual increase from the North to the South in *Synechococcus*, picoeucaryotes, nanoeukaryotes and cryptophytes, mainly concentrated in the Gulf of Gabès, except for *Prochloroccocus* more abundant in northern coast (Khammeri et al., 2020). Although prokaryotic structure and dynamics have been well studied in the North Western and Eastern Mediterranean Sea (Zaballos et al., 2006; Laghdass et al., 2012; Hazan et al., 2018; Haber et al., 2020), the diversity of planktonic prokaryotes, especially heterotrophic prokaryotes, has not yet been studied in the South Mediterranean Sea (including the Gabès Gulf), despite their potential important role in biogeochemical cycles.

In this study, we evaluated for the first time the diversity and composition of prokaryotic communities in seawater samples collected along the Tunisian coast subjected to physicochemical gradients (from the North to the South), in order to understand how environmental variables, affect both archaeal and bacterial community structure. The first step to describe the microbial communities was to determine the diversity and relative abundances of different phylogenetic groups using Illumina sequencing of 16S rRNA genes. Then, we examined the relationships between the prokaryotic community composition/distribution and the water physicochemical characteristics (i.e., salinity, nutrients, physical properties, chlorophyll content). Finally, we investigate the relationships between the dominant microbial groups to highlight their potential ecological role in this specific area of the Mediterranean Sea.

## Materials and Methods

### Sample Collection

**FIGURE 1:**
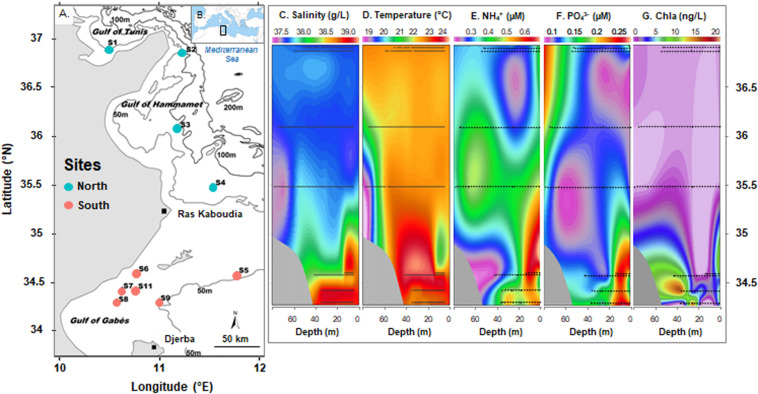
Sampling sites along the Tunisian coast during INCOMMET cruise **(A)** in the South Mediterranean Sea **(B)** with vertical profiles of salinity **(C)**, temperature **(D)**, ammonium **(E)**, orthophosphate **(F)**, and chlorophyll *a* (Chla) **(G)** concentrations along the section defined by the station latitude from the Gulf of Tunis to the Gulf of Gabès (modified from Khammeri et al., 2020). Stations are indicated by blue or red circles (for North or South bays) on map. Stations and sample depth are indicated by line in vertical profiles.

Seawater samples (*n* = 20) were collected at two depths (1 m below the surface and 2 to 3 m above the bottom) from 10 stations along the eastern coast of Tunisia (South Mediterranean Sea, Eastern Mediterranean basin; Figures 1A,B) in November 2013 during the INCOMMET cruise aboard the *N/O Hannibal*. Samples analyzed in this study were selected out of the 30 collected from 11 sites previously described in an ultraphytoplankton community study (Khammeri et al., 2020). Surface (S) and bottom (B) waters were collected using 12-L Niskin bottles fitted on a Rosette sampler equipped with conductivity, temperature, and depth (CTD) sensors (SBE 9, Sea-Bird Electronics) that led to the salinity and temperature data reported in the Figures 1C,D.

Sampling sites were in different bays along the Tunisian coast from North (S1, S2, S3, and S4, located in the Gulf of Tunis and the Gulf of Hammamet) to South (S5, S6, S7, S8, S9, and S11, located in the Gulf of Gabès). These areas are subjected to different physical water properties: the Modified Atlantic Water mass was reported along the northeastern coasts whereas the saltier and warmer Modified Mediterranean Water (MMW) was detected in the Gulf of Gabès (Khammeri et al., 2020). Study area and methodologies used to collect and analyze environmental samples have been described in detail by Khammeri et al. (2020). Water biophysicochemical parameters, such as salinity, temperature, inorganic nutrients (i.e., nitrite: NO_2_^–^, nitrate: NO_3_^–^, ammonium: NH_4_^+^ (Figure 1E), orthophosphate: PO_4_^3–^ (Figure 1F), silicate: Si(OH)_4_, chlorophyll *a* (Chla) (Figure 1G) and planktonic population densities derived by flow-cytometry (i.e., *Synechococcus*, *Prochlorococcus*, pico-, and nano-eukaryotes) have been reported in Table 1.

**TABLE 1:** Physicochemical parameters for seawater samples collected along the Tunisian coast in November 2013.

Samples	Location	Coordinates	Chla (ng/L)	Depth (m)	Density (mg/m^3^)	Tempera-ture (°C)	Salinity (g/L)	NO_2_^–^ (μM)	NO_3_^–^ (μM)	NH_4_^+^ (μM)	PO_4_^3–^ (μM)	NT (μM)	PT (μM)	Si(OH)_4_ (μM)
S1B	Tunis Gulf^1^	36.90 N 10.49 E	0	52	26.00	22.73	37.62	0.26	0.89	0.32	0.13	9.95	3.57	1.81
S2B	Hammamet Gulf^1^	36.80 N 11.23 E	0	59	26.05	22.68	37.66	0.11	1.13	0.33	0.07	9.38	3.39	2.59
S2S	Hammamet Gulf^1^	36.87 N 11.23 E	0	1	26.01	22.69	37.62	0.23	0.71	0.45	0.11	8.82	3.35	3.38
S3B	Hammamet Gulf^1^	36.09 N 11.18 E	0	74	26.30	21.53	37.55	0.06	1.16	0.37	0.17	8.99	2.57	1.44
S3S	Hammamet Gulf^1^	36.09 N 11.18 E	0	1	26.03	22.56	37.60	0.10	0.79	0.37	0.10	7.85	2.52	1.66
S4B	Hammamet Gulf^1^	35.48 N 11.54 E	0	78	26.94	18.85	37.45	0.10	1.09	0.35	0.14	8.69	3.13	2.46
S4S	Hammamet Gulf^1^	35.48 N 11.54 E	0	78	26.94	18.85	37.45	0.10	1.09	0.35	0.14	8.69	3.13	2.46
S5B	Gabès Gulf^2^	34.58 N 11.79 E	10.2	42	26.10	24.19	38.32	0.10	1.14	0.22	0.13	8.62	3.14	2.03
S5S	Gabès Gulf^2^	34.58 N 11.79 E	0	1	26.11	24.18	38.32	0.14	0.79	0.27	0.11	9.15	3.06	2.67
S6B	Gabès Gulf^2^	34.60 N 10.78 E	0	13	27.32	22.02	39.09	0.25	0.77	0.63	0.24	9.01	3.46	2.21
S6S	Gabès Gulf^2^	34.60 N 10.78 E	13.4	1	27.32	22.05	39.09	0.25	0.93	0.65	0.27	8.78	3.23	3.00
S7B	Gabès Gulf^2^	34.42 N 10.63 E	0	26	26.75	23.67	38.97	0.16	0.90	0.49	0.18	8.97	4.12	3.02
S7S	Gabès Gulf^2^	34.42 N 10.63 E	0	1	26.75	23.68	38.97	0.17	1.10	0.43	0.18	8.26	3.18	1.20
S8B	Gabès Gulf^2^	34.30 N 10.58 E	0	33	26.94	23.12	39.01	0.25	0.57	0.53	0.17	8.40	3.13	3.87
S8S	Gabès Gulf^2^	34.30 N 10.58 E	13.2	1	26.87	23.31	38.99	0.26	0.66	0.67	0.25	8.99	3.84	3.64
S9B	Gabès Gulf^2^	34.31 N 11.07 E	17.2	25	27.01	22.63	38.91	0.11	0.67	0.49	0.13	8.95	4.04	3.91
S9S	Gabès Gulf^2^	34.31 N 11.07 E	6.2	1	26.82	22.95	38.78	0.14	0.64	0.37	0.17	9.82	3.69	3.61
S11B	Gabès Gulf^2^	34.43 N 10.77 E	18.2	35	26.96	23.03	39.00	0.08	0.76	0.47	0.12	9.54	4.14	3.32
S11S	Gabès Gulf^2^	34.43 N 10.77 E	14.0	1	26.87	23.29	38.98	0.11	0.96	0.52	0.14	8.69	4.33	4.61

*^1^Tunisian northern bays.*

*^2^Tunisian southern bay.*

For molecular diversity studies of prokaryotic communities, 3L seawater samples were first prefiltered through a 20-μm pore size mesh, then filtered on 2.7-μm pore size, 47-mm-diameter, GF/D filters, to remove large organisms. Picoplanktonic cells were subsequently collected on 0.2-μm pore size, 47-mm-diameter polyethersulfone (PES) filters before being transferred into cryovial tubes containing 3 mL of filtered (0.2-μm) lysis buffer (0.75 M sucrose, 50 mM Tris-HCl and 40 mM EDTA, pH = 8; Massana et al., 2004). Cryovials were immediately frozen in liquid nitrogen.

### DNA Extraction, PCR, and Sequencing Analyses of 16S rRNA Gene Fragments

DNA extraction from 0.2-μm filters was carried out using a phenol/chloroform protocol detailed by Bellaaj-Zouari et al. (2018). The quality and concentration of DNA extracts was measured using a Nanodrop spectrophotometer (Nano Drop 2000 Thermo). DNA samples were stored at −80°C.

Bacterial and archaeal 16S rRNA gene V4 variable regions were amplified by PCR using the Pro341F/Pro805R prokaryotic universal primer set (Takahashi et al., 2014), with barcode on the forward primer, as previously described by Dowd et al. (2008), and were sequenced by the MiSeq Illumina (paired-end 2 × 300 bp) platform of the Molecular Research Laboratory (TX, United States).

Raw amplicon sequencing data were processed using DADA2 version 1.12.1, a model-based approach for correcting amplicon sequencing errors (Callahan et al., 2016). After the inspection of quality read profiles, the data processing steps includes quality filtering, dereplication, denoising, merging, inference of ASVs (Amplicon Sequence Variants, i.e., the true error-free sequences), and chimera removal. This process generates an error-corrected table of the abundances of ASVs in each sample. Because of low sequence number, one sample (S1S) was removed from the dataset for further analysis. The taxonomic assignment of ASVs were performed using the SILVA 16S rRNA gene reference (release 132) database (Quast et al., 2012). Finally, sub-sampling normalization, alpha and beta diversity were investigated by the Phyloseq and vegan R packages (McMurdie and Holmes, 2013; Oksanen et al., 2016).

Sequences from archaeal MGII ASVs were aligned using Muscle (Edgar, 2004) with related sequences from NCBI databases and sequences of *Thaumarchaeota* (MGI) which were used as outgroup. A phylogenetic tree was built with MEGA7 (Kumar et al., 2016) using the Maximum Likelihood method (Tamura and Nei, 1993). Tree topology confidence was determined by bootstrap analysis on 1000 replicates (Felsenstein, 1985).

Raw sequence data are available in the Sequence Read Archive (SRA) of NCBI under BioProject PRJNA632896, BioSamples SAMN14927758–SAMN14927776.

### Statistical Analyses

Statistical analyses were performed with R version 3.6.0 (R Core Team, 2013) using R Studio environment version 1.2.1335. The alpha diversity was assessed by calculating global richness (number of observed ASVs), Shannon (Shannon and Weaver, 1949) and Simpson (Simpson, 1949) indices. The level of sequencing effort was evaluated by rarefaction curves of the observed richness using the *rarecurve* function implemented in the vegan R package. Principal coordinate analysis (PCoA) of beta diversity based on Bray-Curtis distance matrix (from ASVs) was performed for visualizing the distribution of samples along the Tunisian coast with the contribution of the 25 most abundant ASVs. Samples were categorized based on sampling location (North or South, i.e., Gulf of Gabès) or depth (surface, S, or bottom, B). Difference in the alpha diversity indices, the relative abundance of microbial taxa (phylum/class/order/family/genus) or water physicochemical parameters depending on sampling location or depth were assessed using the non-parametric Kruskal-Wallis test followed by Dunn’s test with Bonferroni correction (according to the results of a Shapiro-Wilk normality test). *P* values of <0.05 were considered as statistically significant differences. A Spearman rank correlation test was chosen to investigate the correlations among abundant bacterial taxa (at genus level >1% of sequences per sample in average) and environmental parameters, and we accepted correlation coefficients (*r*_S_) with associated *p*-values <0.05. Dominant genera were also analyzed by a heatmap based on *Z*-scores and a double hierarchical clustering (Ward method). The relationship between the dominant ASVs (selection of the top 40 ASVs, mean relative abundance >0.5%) and environmental factors was carried out by Canonical correspondence analysis (CCA) with Vegan package. The *bioenv* function in the vegan R package selected the best combination of all environmental factors to explain the spatial patterns in the biological matrix. The CCA function used the ASV abundance matrix (transformed by log1p function) with the best combination of environmental factors. The *envfit* (permutation = 999) function fitted environmental factors onto the ordination for those with a *p*-value <0.05.

## Results

### 16S rRNA Gene Analysis and Alpha Diversity

A total of 20 seawater samples were collected and analyzed for prokaryotic community variation along the Tunisian coast, including 12 samples from the Gulf of Gabès (Table 1). The 16S rRNA gene sequencing of samples resulted in 868,099 raw sequences, which were further reduced to 438,375 after filtering, denoising, merging, and chimera checking. The rarefaction curves reached to asymptote (Supplementary Figure S1), indicating a sufficient sequencing effort to collect the overall prokaryotic diversity from the sampling.

**FIGURE 2:**
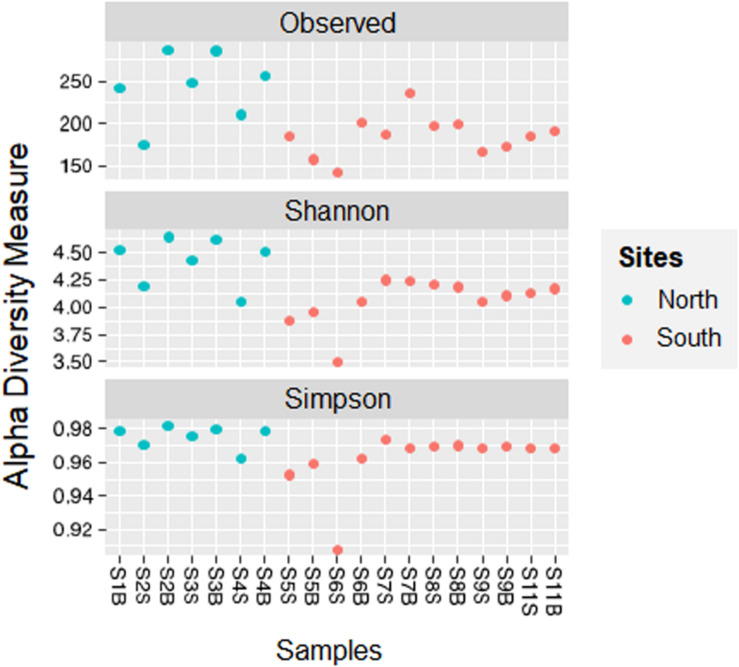
Comparison of alpha diversity indices (Observed, Shannon, Simpson) between sampling sites and location groups (from North or South bays) along Tunisian coast.

The number of reads for each sample is reported in Supplementary Table S1. Across all samples, a total of 1,028 unique ASVs were identified. The number of observed ASVs varied from one sampling site to another, with a maximum (290 ASVs) for the station in the Gulf of Hammamet (North bay) at more than 60 m (S3F) and a minimum (143 ASVs) for the station near Sfax (South bay, Gabès Gulf, S6S). Based on the Simpson (D) and Shannon (H) indices, the microbial diversity in the northern samples (D_North_ = 0.98 ± 0.01; H_North_ = 4.43 ± 0.22) was statistically higher than that in the southern samples (D_South_ = 0.96 ± 0.01; H_South_ = 4.06 ± 0.20; p_D_ = 0.012; p_H_ = 0.016; Figure 2). Regarding all surface (S) and bottom (B) waters group samples, no difference in diversity indices was observed (*p* > 0.05). However, within the northern waters, the diversity indices were higher at the bottom (B) than at the surface (S; p_D_ = 0.049; p_H_ = 0.026).

### Prokaryotic Community Composition

**FIGURE 3:**
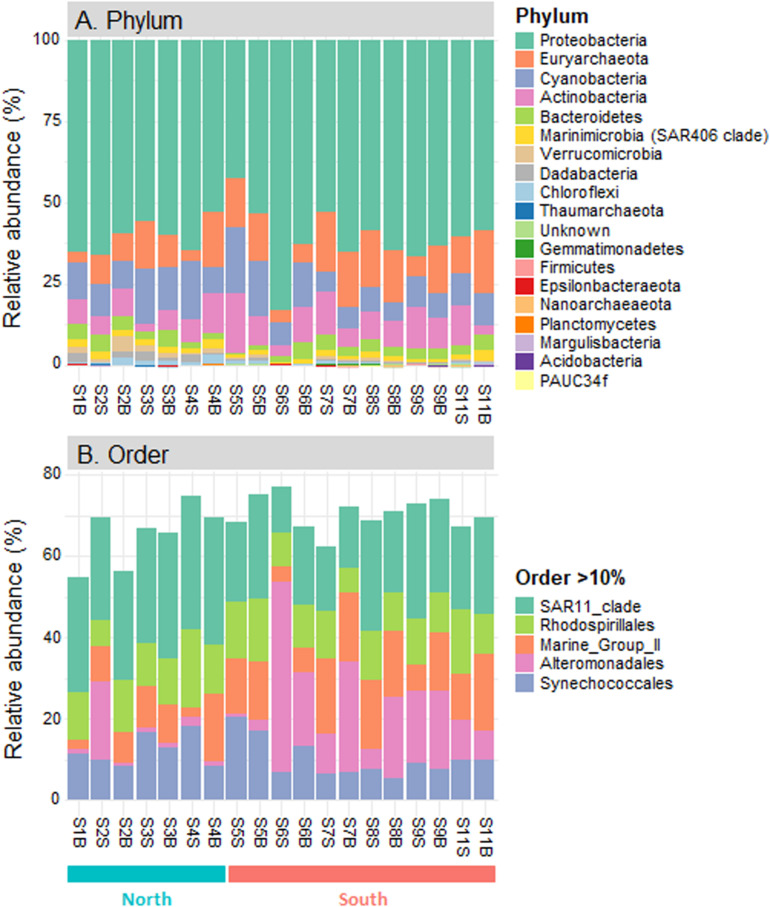
Composition of prokaryotic communities in the seawater samples along the Tunisian coast (South Mediterranean Sea). Relative abundance of all prokaryotic phyla **(A)** and dominant orders (>10% in average, **B**).

Eighteen different phyla were identified across the seawater samples collected along the Tunisian coast, including *Acidobacteria*, *Actinobacteria*, *Bacteroidetes*, *Chloroflexi*, *Cyanobacteria*, *Dadabacteria*, *Epsilonbacteraeota*, *Euryarchaeota*, *Firmicutes*, *Gemmatimonadetes*, *Margulisbacteria*, *Marinimicrobia* (SAR406 clade), *Nanoarchaeaeota*, PAUC34f, *Planctomycetes*, *Proteobacteria*, *Thaumarchaeota*, and *Verrucomicrobia* (Figure 3A). Half of them (nine phyla) were present in all sampling sites and represented more than 99.9% of prokaryotic sequences. Among these nine ubiquitous phyla, *Proteobacteria* was predominant in all samples (60.8 ± 8.0%, 42.8−82.5%), followed by *Cyanobacteria* (10.9 ± 4.4%, 5.8−20.2%) and *Euryarchaeota* (12.0 ± 5.5, 2.8−19.5%; Figure 3A). These three major phyla (each >10% in average) accounted for 84.0% of all prokaryotic sequences. In addition, six other ubiquitous phyla were present at lower proportions, including *Actinobacteria* (8.6 ± 4.0%), *Bacteroidetes* (3.0 ± 1.4), and *Marinimicrobia* (SAR406 clade; 1.6 ± 0.7%), followed by *Verrucomicrobia*, *Chloroflexi* (SAR202 cluster) and *Dadabacteria* (<1% in average). At lower taxonomic ranks, the prokaryotic communities were dominated by five dominant orders (each >10% in average): alphaproteobacterial *Pelagibacterales* (SAR11, 23.9% in average) and *Rhodospirillales* (11.4%), followed by archaeal Marine Group II (MGII, 11.3%), gammaproteobacterial *Alteromonadales* (11.0%) and cyanobacterial *Synechococcales* (10.9%) (Figure 3B).

### Spatial Distribution of Abundant Prokaryotic Taxa

**FIGURE 4:**
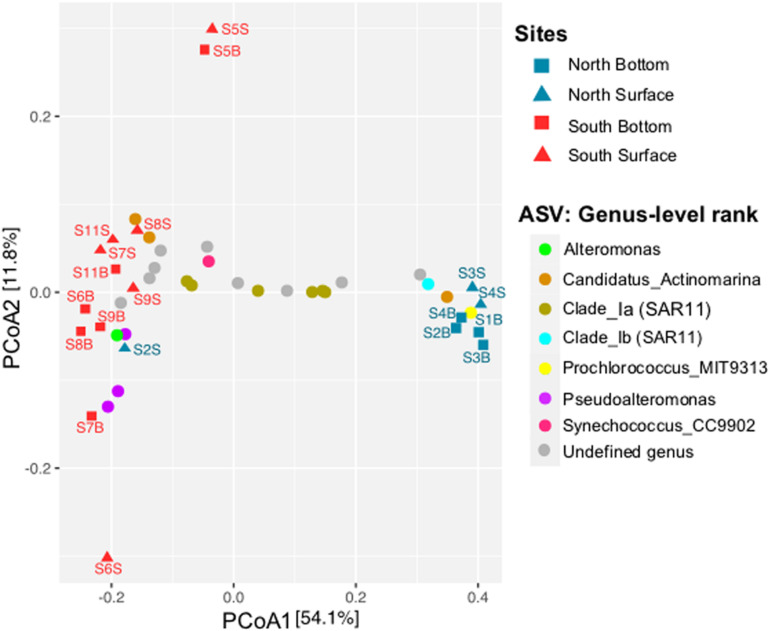
Principal Coordinate Analysis (PCoA) ordination based on Bray-Curtis distance matrix from the prokaryotic community (ASV level) across all seawater samples collected along Tunisian coast. Sample locations are indicated by blue (North) or red (South) symbols (squares or triangles for bottom or surface waters, respectively). The distribution of the 25 most abundant ASVs is indicated by circles of different colors and their taxonomic affiliations are given in the legend at genus-level rank.

The PCoA plot based on the Bray-Curtis distance matrix of the prokaryotic community (ASV level) showed a spatial dichotomy between the North and the South sites (Figure 4). Indeed, the northern samples were ordinated close together (on the right, except for S2S), and separated from the southern samples by the first dimension (PCo1, 54.1% of the total variation). The southern samples (Gulf of Gabès) were relatively grouped together (on the left), but higher variability in the prokaryotic composition of the samples were observed compared to the North. Moreover, the southern samples S5S and S5B (located North of the Gabès Gulf) were mainly separated from the other samples by the second dimension (PCo2, 11.8% of the total variation). Regarding the spatial distribution of samples according to depth, surface (S) vs. bottom (B), no depth-related pattern in the prokaryotic community profiles were identified, regardless of the taxonomic rank (*p* > 0.05).

**FIGURE 5:**
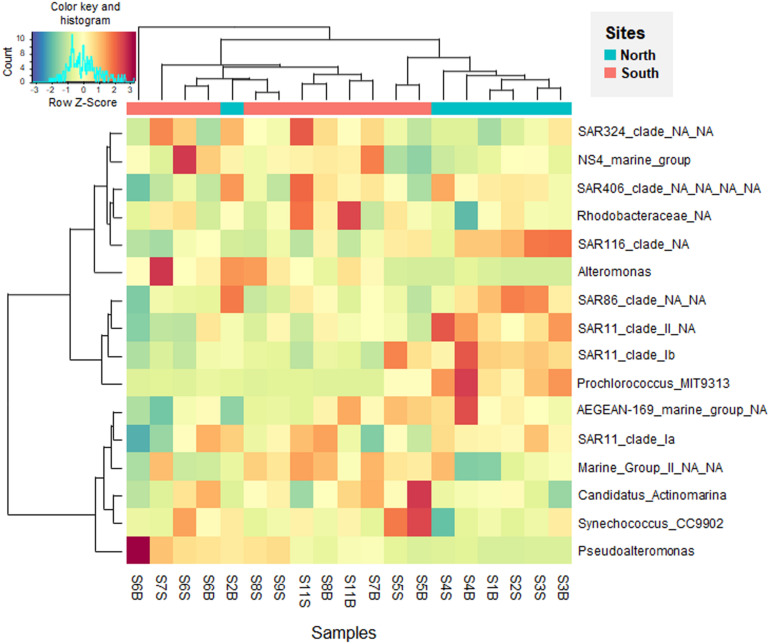
Heatmap visualizing the *Z*-score distribution from the relative abundance of the dominant genera (>1% in average) in the seawater samples along the Tunisian coast (South Mediterranean Sea). The dendogram clusters according to the Bray-Curtis similarity index.

**FIGURE 6:**
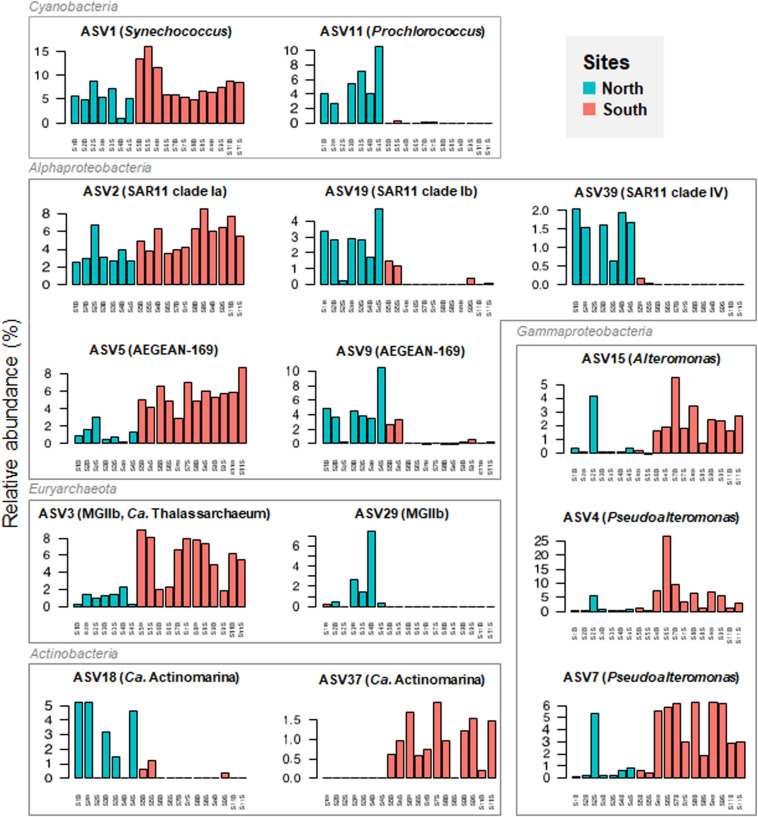
Panel figure showing the relative abundance of dominant ASVs retrieved from the seawater samples collected along Tunisian coast. Representative ASVs of each dominant genus were selected for visualization among top 40 ASVs (mean relative abundance >.5%).

To examine at a finer resolution the differences in the prokaryotic communities within the samples, the contribution of the 25 most abundant ASVs was also visualized on the Bray-Curtis based PCoA plot (Figure 4). A heatmap with dendrogram was constructed using the 16 dominant genera (each >1% in average and accounting for 81.8% of sequences; Figure 5). The distribution of representative ASVs of each dominant genera was visualized by histograms (Figure 6). Both PCoA and heatmap clustering analysis revealed some differences in the prokaryotic relative abundance at ASV and genus levels between the northern or the southern bays, as observed at higher taxonomic ranks, e.g., class, order (Figure 3B).

At the genus level, *Prochlorococcus* and proteobacterial SAR11 Ib and II, SAR86, and SAR116 were more abundant in the North than in the South waters (*p* < 0.005, Figure 5). On the contrary, dominant gammaproteobacterial genera *Pseudoalteromonas* (9.28% in average, dominated by ASVs 4 and 7) and *Alteromonas* (1.69%, dominated by ASV 15), significantly increased in the South (Figures 5 and 6, *p* = 0.002 and *p* = 0.035, respectively). Both *Alteromonas* and the deltaproteobacterial SAR324 (abundantly found in the South) were also detected in high proportion in the northern sample S2B grouped with southern samples (Figure 5), indicating that *Proteobacteria* seems to drive the northern site clustering with southern sites.

At ASV level, *Prochlorococcus* was related to the northern site clustering (in term of abundance, Figure 4), with SAR11 Ib and *Candidatus* Actinomarina, while both *Alteromonas* and *Pseudoalteromonas* ASVs were related to the southern site clustering (Figure 4). Pronounced differences between North and South waters were also observed at ASV level in the relative abundance of dominant archaeal MGIIb (ASV 29), and bacterial ASVs, such as AEGEAN_169 marine ASVs (e.g., ASVs 5 and 9; Figure 6). However, some difference disappeared at higher taxonomic rank (due to the ASV grouping into genus, family, and order). Indeed, no difference between the North and the South (*p* ≥ 0.05) was observed in the distribution of the following major bacterial groups (at the genus level): SAR11 clade Ia (mainly represented by ASV 2), *Synechococcus* (dominated by ASV 1), AEGEAN_169 marine group (*Rhodospirillales*) and *Rhodobacteraceae* (Figures 5, 6). No difference was also globally observed for the major archaeal order (MGII), dominated by ASV3 affiliated to the MGIIb representative, *Candidatus* Thalassoarchaea mediterranii (Supplementary Figure S2).

### Relationships Between Prokaryotic Diversity and Environmental Variables

**FIGURE 7:**
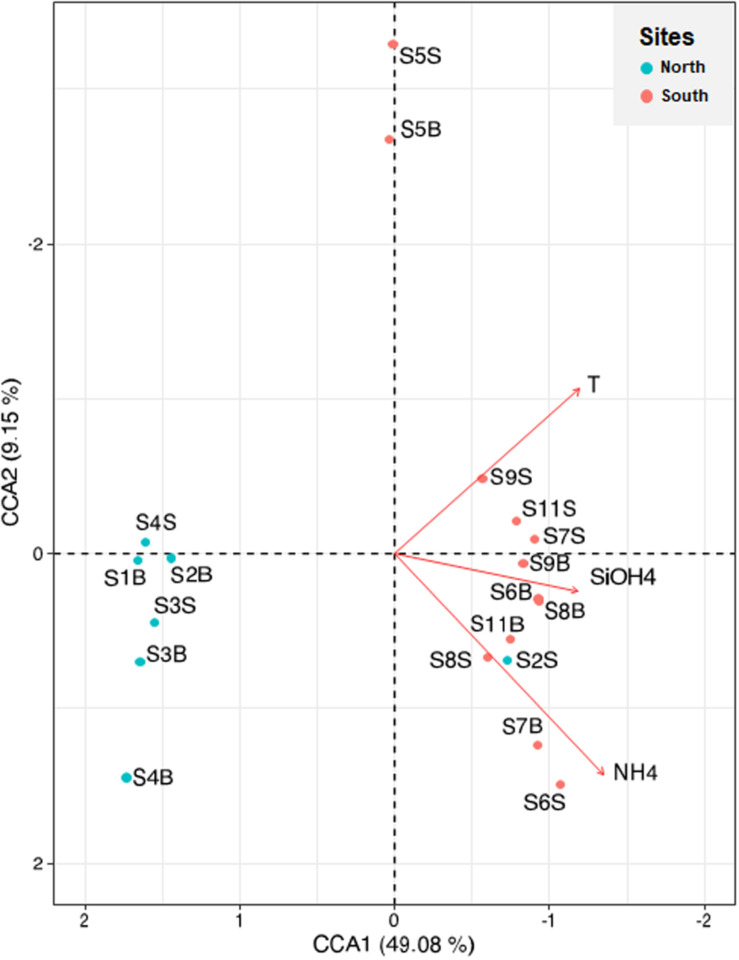
Canonical correspondence analysis visualizing the relationships between physicochemical environmental variables (arrows) and sampling sites (black circles) derived from ASV abundance data. Significant environmental variables are displayed (*p* < 0.05). S1 to S4 sample originated from North Tunisian coast; S5 to S11 samples originate from South Tunisian coast. B and S mean Bottom and Surface waters, respectively.

Canonical correspondence analysis was used to identify the biological and physicochemical environmental parameters that could influence prokaryotic composition in the coastal Tunisian waters (Figure 7). The first two constrained axes explained 49% (CCA1) and 9% (CCA2) of the total inertia (Figure 7). The first axis mostly separated the northern sites from the southern sites, while the second axis separated only the S5 site (South) from the others. The CCA analysis indicated that the prokaryotic composition was significantly related to three environmental factors including NH_4_^+^ (*p* = 0.049), temperature (*p* = 0.012) and silicates (*p* = 0.003). The highest levels of these three parameters were found at the southern sites (excepted S5 and S2B samples) (Table 1). The salinity parameter, which was not plotted, reaches the limit of significance (*p* = 0.052), and PO_4_^3–^ was also not significant (*p* < 0.05).

A Spearman’s rank correlation analysis was also used to examine the relationships between the microbial diversity mainly represented by the abundant genera (>1%) and the environmental variables (Supplementary Table S2). Five environmental variables (Chla, density, NH_4_^+^, salinity, temperature) increased significantly in the southern samples (*p* < 0.05), with a more marked rise in salinity (*p* < 0.0001) and no significant correlation regarding the abundant *Synechococcus* and SAR11 clade Ia (*p* > 0.05; Supplementary Table S2). On the contrary, the gammaproteobacterial *Alteromonas* and *Pseudoalteromonas* proportions (*r* = 0.83, *p* < 0.05) were positively correlated with salinity (*r* = 0.55 and 0.70, respectively, *p* < 0.05), NH_4_^+^ (*r* = 0.66 and 0.75, respectively, *p* < 0.05), PO_4_^3–^ and water densities (for *Pseudoalteromonas*, *r* = 0.57 and 0.63, respectively, *p* < 0.05), found in high levels in the Gabès Gulf waters. Rather, these *Alteromonadales* were negatively correlated with the levels of SAR11 subclades Ib and II and *Prochlorococcus* (*r* = −0.74–0.77, *p* < 0.05; Supplementary Table S3), which were negatively correlated with salinity (*r* = −0.81–0.92, *p* < 0.05), NH_4_^+^ (*r* = −0.79–0.86, *p* < 0.05), PT, PO_4_^3–^ and water densities (*r* = −0.50–0.55 and *r* = −0.52–0.60, respectively, *p* < 0.05; Supplementary Table S2). Both SAR11 Ib and II were positively associated with *Prochloroccocus* (*r* = −0.82–0.83, *p* < 0.05; Supplementary Table S3), which were positively correlated with depth (*r* = −0.50–0.55, *p* < 0.05; Supplementary Table S2). *Rhodobacteraceae* was the only one to be positively correlated with the Chla levels (*r* = 0.63, *p* < 0.05), *Synechococcus* abundance, obtained by flow cytometry; *r* = 0.82, *p* < 0.05 (Supplementary Table S4) and *Synechococcus* relative abundance obtained by 16S rRNA gene sequencing analysis; *r* = 0.59, *p* < 0.05, while the archaeal MGII group was the only one to be positively correlated with temperature (*r* = 0.59, *p* < 0.05). Shannon and Simpson indices were inversely related to *Synechococcus* and *Prochlorococcus* proportions (*r* = −0.52–0.57, *p* < 0.05; Supplementary Table S4).

## Discussion

To date, diversity studies on prokaryotic communities using molecular approaches have received limited attention in coastal waters of the South Mediterranean Sea, despite the presence of marked physicochemical gradients and ecological threat caused by climatic changes and anthropogenic impacts, such as intensive agricultural and industrial activities, as well as international shipping and tourism development. To fill this gap, the planktonic prokaryotic diversity was investigated along the Tunisian coast. This study highlighted changes in the distribution of some microbial taxa from the northern to the southern waters depending on the following environmental variables: Chla, density, nutrients, salinity, and temperature. However, the absence of a depth-related variation in the overall prokaryotic community structure could be explained by the shallowness of the water column sampled and the absence of water stratification in all stations except the deepest station S4 (Figure 1). Indeed, the hydrodynamic conditions measured during the cruise are typically associated with the end of summer-fall stratification in the Mediterranean Sea and the beginning of winter mixing (Bel Hassen et al., 2009b; Bellaaj-Zouari et al., 2018).

The order *Pelagibacteriales* (SAR11) is the most relatively abundant prokaryotic component thriving in coastal Tunisian seawaters, in agreement with previous studies on other Mediterranean Sea areas (Zaballos et al., 2006; Alonso-Sáez et al., 2007; Feingersch et al., 2010; Grote et al., 2012; Laghdass et al., 2012; Viklund et al., 2013; Estrada and Vaqué, 2014; Haber et al., 2020). It was dominated by the genus *Pelagibacter* (SAR11 subclade Ia; 15.3% of the prokaryotes), represented by very small cultivated species adapted to nutrient-limited conditions, and involved in the remineralization of low molecular weight organic matter (Morris et al., 2002; Rappé et al., 2002; Giovannoni et al., 2005; Brown et al., 2012). Along the Tunisian coast, no significant spatial difference in the *Pelagibacter* proportion was observed, while the occurrences of some SAR11 subclades (Ib, II, and IV) decreased significantly from North to South (Gulf of Gabès). Since SAR11 contributed up to 25% of total prokaryotes in Tunisian waters, the distribution changes within the subclades may have important implications on the biogeochemical cycles. Despite its ubiquity and abundance, the distribution and activity of the different SAR11 subclades depending on environmental variables were still unclear, as previously reported in the northwestern Mediterranean surface waters (Laghdass et al., 2012; Salter et al., 2015). In our study, the levels of SAR11 Ib and II were negatively correlated to salinity (*r* = −0.85 and −0.76, respectively) and SAR11 Ib was also negatively correlated to NH_4_^+^ (*r* = −0.79) and PO_4_^3–^ (*r* = −0.64), suggesting that the low proportions of these SAR11 subclades in the Gabes Gulf may be due to its salty, and nutrient-rich waters. Unlike a previous study conducted along a halocline in the Balic Sea (Herlemann et al., 2014), these subclades have not been substituted in the Gabes Gulf by others oligotrophic SAR11 subclades, which prefer low-nutrient environments (Giovannoni, 2017).

Pronounced differences in the cyanobacterial relative abundances were also observed between the northern and the southern Tunisian seawaters. Changes in the *Prochlorococcus* distribution along the Tunisian coast were consistent with previous flow cytometry data obtained according to the described water masses (Khammeri et al., 2020). The *Prochlorococcus* abundance was high in the North corresponding to the MAW and considerably decreased in the shallow, salty, and nutrient-rich waters of the South (where they were absent in the samples S6F, S6S, S8S, S8F, and S11S) corresponding to the MMW. Here, *Prochlorococcus* proportion was negatively correlated to NH_4_^+^ (*r* = −0.86), PO_4_^3–^ (*r* = −0.55) and salinity (*r* = −0.92), found in high levels in the Gabès Gulf waters. Indeed, *Prochlorococcus* preferentially thrives in oligotrophic (nutrient-poor), warm, and stratified waters, usually in summer/fall and is generally absent from eutrophic areas (Chisholm et al., 1988; Partensky et al., 1999b; Durand et al., 2001; Mella-Flores et al., 2012). The highest proportions of the ubiquitous *Synechococcus* observed in southern samples (S5S, S5B, and S6S), suggesting that *Synechococcus* is more adapted than *Prochlorococcus* to the hydrodynamic and nutrient-rich conditions of the Gabès Gulf. Indeed, *Synechococcus* genus is known to be widely distributed, but it is most abundant in well-lit and nutrient-rich waters, usually in a well-mixed coastal water column (Waterbury et al., 1979; Partensky et al., 1999a; Zwirglmaier et al., 2008). Similar trend was also observed along the eastern coast of the Adriatic Sea located North of the Eastern Mediterranean basin (Šantić et al., 2011) and in other marine areas (Flombaum et al., 2013; van den Engh et al., 2017).

The gammaproteobacterial order *Alteromonadales* was more prevalent in the southern waters (Gabès Gulf), than in northern waters (except in S2S). Both *Alteromonas* and *Pseudoalteromonas* proportions were positively correlated with salinity, NH_4_^+^, PO_4_^3–^ and water densities, found in high levels in the Gabès Gulf waters. *Pseudoalteromonas* was more abundant than *Alteromonas* in the Gabès Gulf waters, especially in the surface waters S6S (near the Sfax City), where it accounted for 43.81% of the prokaryotes. These typical marine heterotrophs play important roles in the biodegradation of marine organic matter, such as organic nitrogen and phosphorous mineralization, owing to their high production of proteases and extracellular alkaline phosphatases (Thomas et al., 2008; Zhou et al., 2009; Li et al., 2015; Liu and Liu, 2020). Indeed, higher organic phosphorous in the South than in the North (Khammeri et al., 2020) may induce active biodegradation processes by these heterotrophic organisms. Other heterotrophs detected in nutrient-rich Gabès Gulf waters were related to *Deltaproteobacteria* (SAR324 clade, also involved in sulfur oxidation and carbon fixation, Sheik et al., 2014), as well as to *Actinobacteria* (Ca. *Actinomarina*) and *Bacteroidetes* (NS4 marine group) also known as key players of the organic matter processing (i.e., transport and degradation) in oceans (Kirchman, 2002; Ghai et al., 2013; Anandan et al., 2016). Several studies have reported changes in microbial community composition with a dominance of heterotrophs in marine mesocosms and Mediterranean ecosystems enriched with minerals and nutrients, such as coastal urbanized areas (Allers et al., 2007; Rekik et al., 2014; Richa et al., 2017). The Gulf of Gabès is impacted by industrial and municipal discharges that increase toxic metals (such as cadmium) and nutrients (such as PO_4_^3–^) in marine environment, that cause seawater pollution (e.g., eutrophication) and microbial diversity changes (Zouch et al., 2017, 2018; Chifflet et al., 2019).

Beyond abiotic factors, heterotrophic microorganisms can impact and interact with a wide range of prokaryotic organisms in marine ecosystems. Heterotrophic *Pseudoalteromonas* species exhibit high extracellular activities and antimicrobial properties allowing them to hydrolyze complex molecules and to be extremely competitive for nutrients (Holmström and Kjelleberg, 1999). In our study, the relative abundance of both heterotrophic *Pseudoalteromonas* and *Alteromonas* were negatively correlated with photosynthetic *Prochlorococcus* (*r* = −0.71–0.75), accordingly to previous co-culture findings (Becker et al., 2019). On the contrary, the relative abundance of *Prochlorococcus* was positively correlated with some SAR11 clades (Ib and IV) (*r* = 0.82–0.83), in agreement with the study of Becker et al. (2019) showing that SAR11 grew faster in co-culture with *Prochlorococcus*, likely due to the production and release of glycine betaine by this latter. This result suggests that some SAR11 clades, detected in low abundance in the South, might be also influenced by the *Prochlorococcus* decrease in the Gabès Gulf. The relative abundance of *Rhodobacteraceae* was also positively correlated to *Synechococcus* and chlorophyll *a* (*r* = 0.63–0.71), suggesting a link with phytoplankton biomass, as revealed by a previous study in marine water mesocosms (Allers et al., 2007).

*Archaea* were well represented in all Tunisian coastal seawater samples (>10% of the prokaryotes) and were mainly composed of the candidate order “Poseidoniales” (MGII), as observed in coastal northwest Mediterranean Sea and in surface waters of different marine areas around the world (Galand et al., 2010; Hugoni et al., 2013; Pereira et al., 2019; Rinke et al., 2019; Santoro et al., 2019). In our winter study, the MGIIb group (Candidatus *Thalassarchaeaceae*, represented by the ASVs 3, 21, 29, 40) was more abundant that the MGIIa (represented by the ASVs 28 and 41) in seawaters. These results agree with previous data on the northwest Mediterranean Sea showing MGIIb as a major archaeal group in winter, while MGIIa predominated in summer (Galand et al., 2010; Hugoni et al., 2013; Martin-Cuadrado et al., 2015). Recent MGII genomic analyses provide evidence for a photoheterotrophic lifestyle combining phototrophy via proteorhodopsins with organic matter remineralization (Martin-Cuadrado et al., 2015; Tully, 2019). In shallow Tunisian coastal waters, both MGIII and *Thaumarchaeota* (mainly ammonia-oxidizing *Nitrososphaera*) were detected in low abundance (<1% and <0.1% of the prokaryotes, respectively), in accordance with their known prevalence in deep waters (Techtmann et al., 2015; Santoro et al., 2019). The predominance of MGIII in the surface waters of the Gulf of Hammamet (S3S, 4.56% of the prokaryotes), displaying the lowest level of PO_4_^3–^, may be explained by the presence in its genomes of photolyase genes and phosphonate uptake, which may serve as a phosphorus source in inorganic phosphorus-deficient waters (Santoro et al., 2019). Finally, *Nanoarchaeota* were exclusively found in extremely low proportion (around 0.02%) in the bottom waters of the Gabès Gulf and they are known as small parasitic or symbiotic Archaea (Amils, 2014).

In conclusion, our findings on prokaryotic diversity in seawater samples along the Tunisian coast increase our knowledge on microbial biodiversity and potential ecosystem functioning in the understudied regions of the South Mediterranean Sea. Amplicon sequencing-based prokaryotic community analysis allowed us to identify typical dominant and ubiquitous marine taxa affiliated to phyla *Proteobacteria* (alphaproteobacterial SAR11 clade and gammaproteobacterial *Alteromonadales*), *Cyanobacteria* (*Synechococcales*), and *Euryarchaeota* (“Poseidoniales”). Our comparative analysis between the North and South Tunisian bays showed several changes in the prokaryotic community composition, especially within the SAR11 clade, the order *Alteromonadales* and the order *Synechococcales*. A significant increase in the levels of the genera *Pseudoalteromonas* and *Alteromonas* were observed in the southern waters (Gulf of Gabès), compared to northern bays, and was inversely related to *Prochlorococcus* proportion. These changes may be explained by the difference in physical water properties, mainly temperature and nutrient content and Chla concentrations, in accordance with previous studies in the Mediterranean Sea. It is worthy to continue analyzing the diversity of the microbial community in future investigations toward larger spatial and temporal scales in the South Mediterranean Sea, in order to confirm these microbial biodiversity patterns and better understand the important biogeochemical processes mediated by specific microbial groups in a context of global climatic change and/or human-induced environmental changes.

## Data Availability Statement

The datasets presented in this study can be found in online repositories. The names of the repository/repositories and accession number(s) can be found in the article/Supplementary Material.

## Author Contributions

AB-Z, MB, and YK performed the water sampling during the INCOMMET cruise aboard the *N/O Hannibal*. YK performed core parameter analyses. AB-Z performed DNA extraction. MQ processed the Illumina MiSeq data with the help of FA. MQ wrote the first draft of the manuscript. All authors were involved in the critical revision and approval of the final version.

## Conflict of Interest

The authors declare that the research was conducted in the absence of any commercial or financial relationships that could be construed as a potential conflict of interest.
