# Supplementary material for: Prokaryotic Diversity and Distribution Along Physical and Nutrient Gradients in the Tunisian Coastal Waters (South Mediterranean Sea)
Source: Front Microbiol. 2020 Dec 1;11:593540. doi: 10.3389/fmicb.2020.593540 (PMC7735998; doi:10.3389/fmicb.2020.593540)

Figure S1. Rarefaction curves for the 19 samples showing the diversity detected compared with the predicted total diversity. The x axis represents the number of sequences sampled while the y axis represents a measure of the species richness detected. The legend on the right shows the correspondence between the curves and the samples. For labeling of the samples see Table 1.

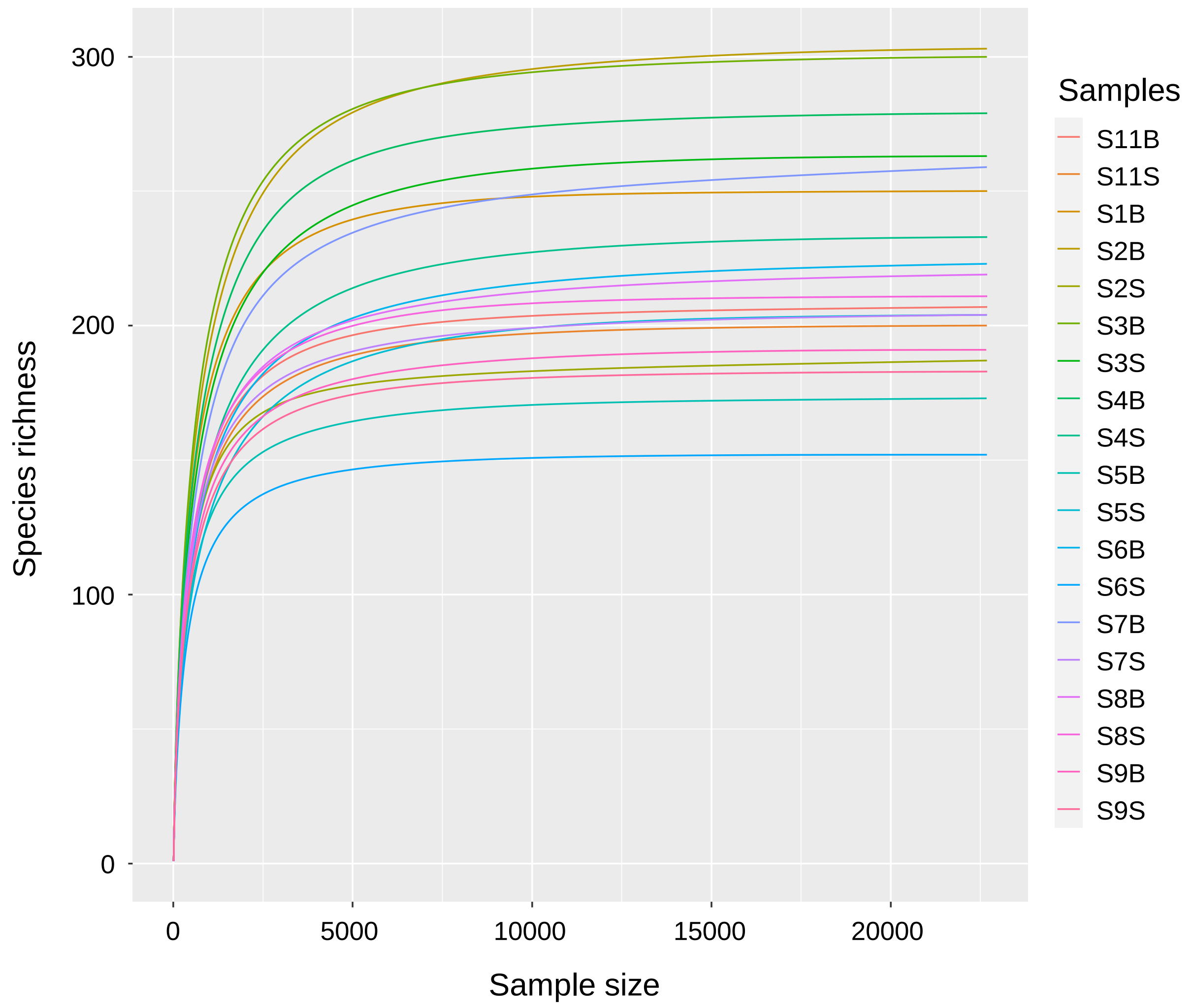

Figure S2. Maximum Likelihood phylogenetic tree based on 389 aligned bp of archaeal MGII 16S rRNA gene sequences. Archaeal MGII ASV sequences originated from the seawater samples collected along Tunisian coast are marked in bold. Circles indicate the relative abundance of these sequences in northern (blue) and southern (red) samples. Other sequences correspond to the closest environmental or representative sequences found by BLAST analyses or reference sequences of the MGII clades indicated in Pereira et al. (2019). MGI sequences (AB019726, AB019723, EU280202) were used as outgroup. Bootstrap values higher than 50% (based on 1000 replicates) are shown at branch nodes. Scale bar: 0.05 substitution per site.

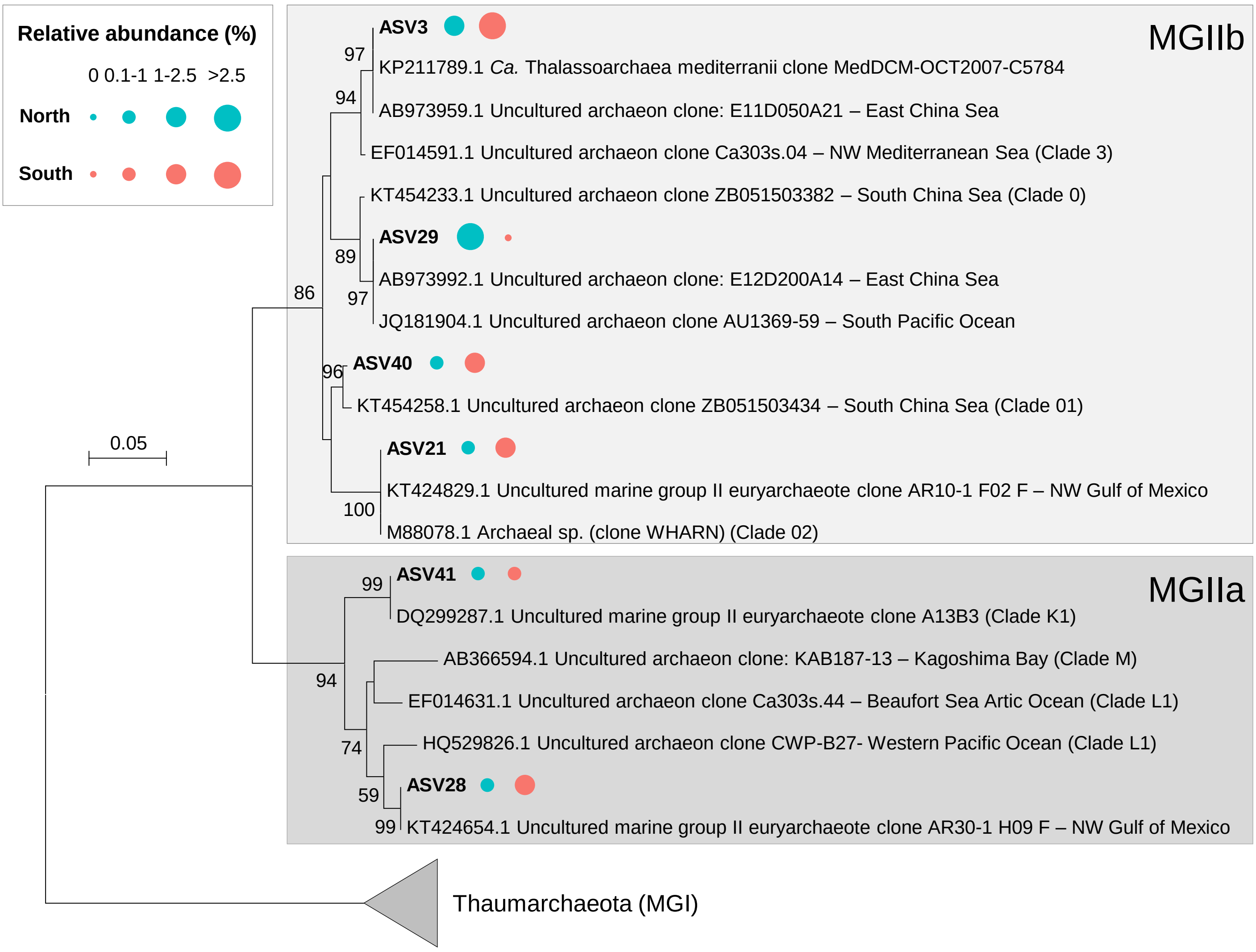

Supplement: Supplementary file 1 [file Data_Sheet_1.PDF]
