# Supplementary material for: Prokaryotic Diversity and Distribution Along Physical and Nutrient Gradients in the Tunisian Coastal Waters (South Mediterranean Sea)
Source: Front Microbiol. 2020 Dec 1;11:593540. doi: 10.3389/fmicb.2020.593540 (PMC7735998; doi:10.3389/fmicb.2020.593540)
Supplement: Supplementary file 2 [file Data_Sheet_2.pdf]

Table S1. Number of sequences analyzed and alpha diversity indices.

Samples	Sequence numbers					Final retained (%)	Alpha diversity indices		
	Input	Filtered	Denoised	Merged	Non chimeric		Observed	Shannon	Simpson
S1B	39969	25933	24492	21155	19090	47.76	244	4.51	0.98
S2B	59335	38640	36902	32018	28954	48.80	290	4.67	0.98
S2S	29439	18151	17531	15980	15668	53.22	176	4.20	0.97
S3B	62389	39677	37929	32906	28144	45.11	290	4.64	0.98
S3S	52669	34208	32754	29021	25962	49.29	251	4.43	0.98
S4B	62376	38741	37176	32459	27539	44.15	259	4.52	0.98
S4S	47852	32362	31461	29113	26594	55.58	214	4.06	0.96
S5B	33165	20947	20346	18715	18177	54.81	160	3.95	0.96
S5S	63430	39919	38485	34835	30881	48.69	191	3.89	0.95
S6B	44695	28465	27861	26004	25286	56.57	211	4.04	0.96
S6S	24931	15811	15148	13442	13017	52.21	143	3.50	0.91
S7B	60280	37020	35908	32473	30888	51.24	241	4.25	0.97
S7S	39147	24028	23148	20940	20389	52.08	189	4.23	0.97
S8B	56574	34327	33194	29075	26799	47.37	205	4.18	0.97
S8S	40684	25942	24870	21688	19924	48.97	197	4.20	0.97
S9B	41874	26237	25419	23200	22564	53.89	175	4.10	0.97
S9S	35094	22430	21742	19753	18925	53.93	169	4.08	0.97
S11B	31321	19053	18741	17323	16888	53.92	193	4.17	0.97
S11S	42875	26898	26113	23608	22686	52.91	188	4.15	0.97

Table S2. Spearman's rank correlation coefficients between the physicochemical parameters and the relative abundance of dominant genera (>1%) observed in seawater samples collected along the Tunisian coast in November 2013. Values in bold are significant at $p \leq 0.05$.

Genus/Variables	Chla	Depth	Density	Salinity	Temperature	NO_2^-	NO_3^-	NH_4^+	PO_4^{3-}	NT	PT	$Si(OH)_4$
Synechococcus_CC9902	0.32	-0.47	-0.14	0.14	0.30	-0.05	-0.26	-0.08	-0.32	0.18	0.11	0.05
SAR11_Clade_Ia	0.09	0.25	-0.10	-0.38	-0.33	-0.25	-0.22	-0.10	-0.17	0.46	0.12	0.07
Marine_Group_II	0.21	-0.10	0.11	0.28	**0.58**	-0.11	-0.11	0.19	0.07	-0.25	0.16	0.18
Pseudoalteromonas	0.34	-0.43	**0.63**	**0.70**	0.11	0.43	-0.45	**0.75**	**0.57**	-0.15	0.41	0.49
AEGEAN-169 group	-0.03	0.26	-0.18	-0.38	0.07	-0.28	0.45	**-0.54**	-0.19	-0.04	-0.15	-0.20
Prochlorococcus_MIT9313	-0.42	**0.50**	**-0.52**	**-0.92**	-0.36	**-0.60**	0.41	**-0.86**	**-0.55**	0.02	**-0.55**	**-0.56**
SAR11_Clade_II	-0.21	0.37	-0.30	**-0.76**	-0.44	-0.38	0.20	-0.41	-0.18	-0.09	-0.25	-0.29
Candidatus Actinomarina	-0.03	-0.18	0.09	0.11	0.32	0.17	-0.02	-0.14	0.11	0.02	0.03	0.09
Alteromonas	0.28	-0.42	0.30	**0.55**	0.26	0.49	**-0.50**	**0.66**	0.28	-0.12	**0.58**	**0.65**
SAR11_Clade_Ib	-0.42	**0.51**	**-0.60**	**-0.85**	-0.21	-0.41	0.38	**-0.79**	**-0.54**	0.00	**-0.51**	-0.38
Rhodobacteraceae	**0.63**	-0.25	0.22	0.42	0.32	0.07	-0.09	0.27	0.02	0.20	**0.54**	0.36
NS4_marine_group	0.32	-0.47	0.28	0.49	-0.04	0.27	-0.31	**0.58**	0.31	0.17	**0.53**	0.24
SAR86_clade	-0.33	0.36	**-0.53**	**-0.52**	-0.27	-0.29	0.33	-0.32	-0.37	0.20	-0.09	-0.49
SAR324_clade	0.09	-0.19	0.15	0.32	0.19	-0.07	-0.21	0.49	0.02	-0.23	0.31	0.15
SAR11_cladeIV	-0.46	**0.55**	-0.40	**-0.81**	-0.35	-0.43	**0.52**	**-0.80**	-0.32	0.05	**-0.50**	**-0.57**
SAR406_clade	-0.19	0.40	-0.32	-0.37	-0.17	-0.27	0.23	-0.21	-0.27	0.10	-0.05	-0.32

Table S3. Spearman's rank correlation coefficients between the relative abundance of dominant genera observed in seawater samples collected along the Tunisian coast in 2013. Values in bold are significant at $p \leq 0.05$.

Variables	Synec. CC9902	SAR11 Clad_Ia	MGII	Pseudoalt.	AEGEAN-169	Proc. MIT9313	SAR11 Clad_II	Cand. Actinomarina	Alteromonas	SAR11 Clad_Ib	Rhodobacteraceae	NS4_marine_group	SAR86_clade	SAR11_cladeIV	SAR406_clade
Synechococcus_CC9902	**1.00**	0.10	-0.09	0.03	0.07	-0.03	-0.32	0.09	-0.05	-0.01	**0.59**	0.16	0.00	-0.15	-0.08
SAR11_Clade_Ia	0.10	**1.00**	-0.12	-0.38	0.12	0.35	**0.59**	-0.20	-0.28	0.32	-0.03	0.15	**0.53**	0.28	**0.62**
Marine_Group_II	-0.09	-0.12	**1.00**	0.15	-0.16	-0.29	-0.17	0.08	0.14	-0.37	0.22	-0.06	-0.15	-0.37	0.20
Pseudoalteromonas	0.03	-0.38	0.15	**1.00**	**-0.59**	**-0.75**	**-0.58**	0.04	**0.83**	**-0.74**	0.22	0.45	**-0.60**	**-0.77**	**-0.50**
AEGEAN-169_marine_group	0.07	0.12	-0.16	**-0.59**	**1.00**	0.39	0.38	**0.52**	**-0.54**	**0.51**	0.09	-0.29	0.03	**0.70**	0.04
Prochlorococcus_MIT9313	-0.03	0.35	-0.29	**-0.75**	0.39	**1.00**	**0.68**	-0.15	**-0.71**	**0.82**	-0.44	**-0.57**	0.46	**0.83**	0.30
SAR11_Clade_II	-0.32	**0.59**	-0.17	**-0.58**	0.38	**0.68**	**1.00**	-0.06	-0.44	**0.58**	**-0.46**	-0.14	**0.46**	**0.70**	0.39
Candidatus_Actinomarina	0.09	-0.20	0.08	0.04	**0.52**	-0.15	-0.06	**1.00**	-0.02	-0.05	0.12	0.18	-0.45	0.27	-0.29
Alteromonas	-0.05	-0.28	0.14	**0.83**	**-0.54**	**-0.71**	-0.44	-0.02	**1.00**	**-0.61**	0.10	**0.49**	-0.38	**-0.76**	-0.34
SAR11_Clade_Ib	-0.01	0.32	-0.37	**-0.74**	**0.51**	**0.82**	**0.58**	-0.05	**-0.61**	**1.00**	-0.31	**-0.60**	0.45	**0.81**	0.21
Rhodobacteraceae	**0.59**	-0.03	0.22	0.22	0.09	-0.44	**-0.46**	0.12	0.10	-0.31	**1.00**	0.16	-0.19	-0.37	-0.04
NS4_marine_group	0.16	0.15	-0.06	0.45	-0.29	**-0.57**	-0.14	0.18	**0.49**	**-0.60**	0.16	**1.00**	0.06	-0.42	0.06
SAR86_clade	0.00	**0.53**	-0.15	**-0.60**	0.03	0.46	**0.46**	-0.45	-0.38	0.45	-0.19	0.06	**1.00**	0.36	**0.77**
SAR11_cladeIV	-0.15	0.28	-0.37	**-0.77**	**0.70**	**0.83**	**0.70**	0.27	**-0.76**	**0.81**	-0.37	-0.42	0.36	**1.00**	0.21
Marinimicrobia_(SAR406_clade)	-0.08	**0.62**	0.20	**-0.50**	0.04	0.30	0.39	-0.29	-0.34	0.21	-0.04	0.06	**0.77**	0.21	**1.00**

Table S4. Spearman's rank correlation coefficients between relative abundance of dominant genera and alpha diversity indices (obtained in this study) or biological parameters obtained by flow cytometry from seawater samples collected along the Tunisian coast in 2013. Values in bold are significant at $p \leq 0.05$.

Variables	Observed	*H*	Simpson	Picoeukaryotes*	Synechococcus*	Prochlorococcus*	Nanoeukaryotes*	Cryptophyes*
Synechococcus_CC9902	**-0.51**	**-0.57**	**-0.52**	0.280	**0.819**	0.051	-0.009	0.119
SAR11_Clade_Ia	0.24	0.35	0.35	0.214	-0.011	0.236	-0.243	-0.149
Marine_Group_II	-0.01	0.21	0.08	0.113	-0.001	-0.309	**0.550**	**0.575**
Pseudoalteromonas	**-0.55**	**-0.54**	**-0.55**	0.277	0.004	**-0.743**	**0.559**	**0.581**
AEGEAN-169_marine_group	0.08	-0.01	0.08	0.244	0.268	**0.656**	-0.060	-0.132
Prochlorococcus_MIT9313	0.42	0.26	0.30	-0.307	-0.205	**0.781**	**-0.686**	**-0.776**
SAR11_Clade_II	0.45	**0.55**	**0.67**	-0.017	-0.400	**0.547**	-0.349	**-0.520**
Candidatus_Actinomarina	-0.25	-0.24	-0.04	0.402	0.271	0.032	0.355	0.179
Alteromonas	**-0.48**	-0.30	-0.31	0.144	-0.160	**-0.748**	**0.505**	**0.588**
SAR11_Clade_Ib	0.37	0.24	0.29	-0.454	-0.207	**0.747**	**-0.737**	**-0.653**
Rhodobacteraceae	-0.42	-0.34	-0.38	0.368	**0.713**	-0.171	0.272	**0.459**
NS4_marine_group	-0.31	-0.08	0.07	**0.607**	0.199	**-0.487**	0.422	0.338
SAR86_clade	**0.55**	**0.59**	**0.53**	-0.259	-0.198	0.387	**-0.580**	**-0.482**
SAR11_cladeIV	**0.47**	0.29	0.42	-0.169	-0.153	**0.797**	**-0.550**	**-0.720**
Marinimicrobia (SAR406 clade)	0.44	**0.61**	**0.53**	-0.075	-0.160	0.343	-0.332	-0.226

*Data obtained by flow cytometry from a previous study on ultraphytoplankton (Khammeri et al., 2020)
